# Impact of Quality Improvement on Care of Chronic Obstructive Pulmonary Disease Patients in an Internal Medicine Resident Clinic

**DOI:** 10.3390/healthcare6030088

**Published:** 2018-07-25

**Authors:** Robert M. Burkes, Takudzwa Mkorombindo, Udit Chaddha, Alok Bhatt, Karim El-Kersh, Rodrigo Cavallazzi, Nancy Kubiak

**Affiliations:** 1 Department of Internal Medicine, University of Louisville, 550 S. Jackson Street, ACB 3rd Floor, Louisville, KY 40202, USA; tmkorombindo@uabmc.edu (T.M.); drudit@gmail.com (U.C.); Alok.Bhatt@nyumc.org (A.B.); 2 Division of Pulmonary, Critical Care, and Sleep Medicine Disorders, Department of Internal Medicine, University of Louisville, 550 S. Jackson Street, Pulmonary, Critical Care and Sleep Disorders Medicine Offices, ACB 3rd Floor, Louisville, KY 40202, USA; Karim.elkersh@louisville.edu (K.E.-K.); r0cava01@louisville.edu (R.C.); 3 Department of General Internal Medicine, University of Louisville, Palliative Care, and Medical Education, 550 S. Jackson Street, General Internal Medicine and Palliative Care Offices, ACB 3rd Floor, Louisville, KY 40202, USA; Nancy.Kubiak@louisville.edu

**Keywords:** pulmonary diseases, quality improvement, Medical Education, vaccinations, smoking cessation

## Abstract

Chronic obstructive pulmonary disease (COPD) is a leading cause of morbidity and mortality. Guideline-discordant care of COPD is not uncommon. Further, there is a push to incorporate quality improvement (QI) training into internal medicine (IM) residency curricula. This study compared quality of care of COPD patients in an IM residents’ clinic and a pulmonary fellows’ clinic and, subsequently, the results of a quality improvement program in the residents’ clinic. Pre-intervention rates of quality measure adherence were compared between the IM teaching clinic (*n* = 451) and pulmonary fellows’ clinic (*n* = 177). Patient encounters in the residents’ teaching clinic after quality improvement intervention (*n* = 119) were reviewed and compared with pre-intervention data. Prior to intervention, fellows were significantly more likely to offer smoking cessation counseling (*p* = 0.024) and document spirometry showing airway obstruction (*p* < 0.001). Smoking cessation counseling, pneumococcal vaccination, and diagnosis of COPD by spirometry were targets for QI. A single-cycle, resident-led QI project was initiated. After, residents numerically improved in the utilization of spirometry (66.5% vs. 74.8%) and smoking cessation counseling (81.8% vs. 86.6%), and significantly improved rates of pneumococcal vaccination (*p* = 0.024). One cycle of resident-led QI significantly improved the rates of pneumococcal vaccination, with numerical improvement in other areas of COPD care.

## 1. Introduction

Chronic obstructive pulmonary disease (COPD) is a leading cause of death worldwide, with a prevalence of 10% [1,2]. The burden of COPD and tobacco abuse falls most heavily on those in the lower socioeconomic strata [3]. Further, misdiagnosis of chronic lung conditions is common in this demographic [4]. In light of these findings, it is imperative that residents in academic medical centers that treat underserved populations diagnose COPD accurately and provide high quality care. 

The Accreditation Council for Graduate Medical Education (ACGME) requires residents to obtain skills to analyze patients’ outcomes and “implement changes with the goal of practice improvement” [5]. Despite barriers including time constraints and lack of sufficient faculty with quality improvement (QI) training, projects with resident involvement have been successful at enacting meaningful change in the care of chronic medical conditions [6,7,8,9].

Spirometric testing showing obstructive lung disease, which is required for the diagnosis of COPD [2], is underutilized in the primary care setting. Without the use of spirometric measures, COPD is often misdiagnosed, and patients are treated with inhalers and steroids inappropriately [10,11,12,13,14]. Further, smoking cessation efforts are both efficacious and cost-effective, making cessation counseling an imperative aspect of care provided to COPD patients [15,16]. Also, the reported trend towards prevention of pneumococcal pneumonia and acute exacerbation of COPD with pneumococcal vaccination makes this an important aspect of the longitudinal care of the COPD patient [2].

The goal of this investigation is to evaluate shortcomings in the care of COPD patients in a residents’ clinic and attempt a brief and low-cost intervention to improve these processes of care. This study evaluates the care of COPD patients provided by internal medicine residents as it pertains to guideline-based care and compares process-of-care measures with fellows at the same institution and assesses the feasibility and efficacy of a single round of quality improvement developed by residents and directed at their peers.

## 2. Materials and Methods

### 2.1. Participants

This is a quality improvement study with two distinct phases. An initial retrospective chart review phase included 628 patients (451 seen in the internal medicine residents’ clinic and 177 seen in the pulmonary medicine fellows’ clinic, both at the University of Louisville). For this particular training environment, residents had completed medical school training. Fellows were in pulmonary and critical care specialty training, had completed internal medicine training, and were board eligible/certified to practice general internal medicine (but not yet pulmonary medicine) in the United States. After identifying areas of treatment deficiency, a QI initiative (described below) was implemented from June 2016 to May 2017 (the bulk of one academic year for residents of training programs in the United States). Charts of the subsequent 119 encounters that received a billing code for COPD in the residents’ clinic were reviewed in a prospective fashion.

The charts of patients diagnosed with chronic bronchitis (ICD-9 Code 491), emphysema (ICD-9 Code 492), and/or chronic airway obstruction (ICD-9 Code 496) were selected for review. For the purpose of this study, those with asthma (ICD-9 Code 493) without one of the aforementioned diagnoses, patients with paper charts that were illegible or had poor quality electronic scans, patients seen only once and lost to follow-up, and patients where COPD status was not actively reflected (i.e., COPD or respiratory symptoms never being addressed by provider) in their chart were excluded. Demographics, comorbidities including heart failure and asthma, spirometric data, smoking status, inhaled COPD medications, and vaccination status were collected. Spirometry data was found in either the patient’s inpatient or outpatient chart or from scans of outside records. Each patient’s vaccination record was recorded from inpatient or outpatient charts. Smoking cessation was determined based on the resident clinic note documenting that he or she had provided a smoking cessation intervention to the patient. 

### 2.2. Quality Improvement Intervention

The quality improvement intervention was targeted at all residents (*n* = 72 residents, including this study’s authors, sharing roughly the same size patient panel) in the general internal medicine residency training program at the University of Louisville, using the PDSA model of quality improvement. During the planning stage, the team identified opportunities for improving care by comparing resident-to-fellow performance for COPD guidelines. They crafted a short 15 min PowerPoint-based didactic session discussing current guideline-based care of COPD, data showing the adherence of residents to studied quality measures, and the plan for the future QI intervention. They created easy-to-use index cards to guide correct pneumococcal immunization and remind residents of the need for tobacco cessation counseling and spirometry to confirm diagnosis in patients with COPD. In the Do phase, the presentation was given by a third-year internal medicine resident to both residents and staff in the clinic, and that information was also sent via e-mail correspondence to residents. Residents were also provided weekly verbal reminders of the project. Index cards with an algorithmic approach to pneumococcal vaccination, based on current guidelines, were posted on the clinic’s computers where residents checked-out to attending physicians and did office note documentation. A standing order for pneumococcal immunization was implemented in the clinic, allowing medical assistants to identify and vaccinate COPD patients. In the study phase, information on the effectiveness of the interventions was collected. Since the interventions improved rates, the team opted to continue episodic reminders about the intervention during the Act phase. Because printed index cards were donated, this intervention accrued no cost. The design, presentation, and implementation of the QI initiative was carried out by a group of four internal medicine residents (first four authors).

### 2.3. Outcomes Measured

Our outcomes were the rate at which patients who had a diagnosis of COPD underwent spirometry or pulmonary function testing (PFTs), the rate of pneumococcal vaccination, and documentation of smoking cessation counseling. These were chosen based on perceived importance in the care of COPD patients in the outpatient setting, the wide difference between rates of application in the residents’ and fellows’ clinic, and because these were considered the deficiencies most readily addressable by a QI expert consultant (Kubiak). These measures were compared in the residents’ clinic and the fellows’ clinic retrospectively prior to QI intervention, in order to delineate what was possible in the setting and to establish a baseline for the resident performance. In response to the retrospective findings, the same outcomes were compared in the residents’ clinic before and after the QI intervention in a prospective, un-blinded fashion.

### 2.4. Statistical Analysis

The statistical modeling strategy sought to provide enumeration of adherence to quality measures [17]. We report continuous variables as mean and standard deviation, and categorical variables as frequency and percentage. We used a chi-squared test to compare categorical data and paired *t*-tests for continuous data. We considered a *p*-value less than 0.05 as statistically significant. Because each quality measure was assessed individually and not assumed to be associated with demographic or clinical aspects of the patient cohort, multivariate modeling was not performed. Statistical analysis was performed using Stata 10 (Stata Corp., College Station, TX, USA) software. The publication of this study, including the waiver of consent, was deemed exempt by the institutional review board at the University of Louisville (IRB: 15.0243).

## 3. Results

Prior to QI intervention, of the 628 charts reviewed, 451 patients were seen in the residents’ clinic and 177 patients in the pulmonary fellows’ clinic. The charts of the subsequent 119 patients seen in the residents’ clinic after QI intervention were reviewed after QI intervention. When comparing pre-intervention residents’ clinic to fellows’ clinic, patients seen in the fellows’ clinic were more likely to be female (*p* = 0.039), have a lower forced-expiratory-volume-in-one-second to forced vital capacity ratio (FEV1/FVC) (*p* < 0.001), have a lower percent-predicted forced-expiratory-volume-in-one-second (FEV1) (*p* < 0.001), greater percent predicted residual volume (*p* = 0.006), and lower percent-predicted diffusion capacity (*p* < 0.001). Fellows’ clinic patients were more likely to have been prescribed a long-acting muscarinic agent, inhaled corticosteroids, or long-acting beta agonists (*p* < 0.001 for each class of medication), and to have been prescribed home oxygen for chronic hypoxic respiratory failure (*p* < 0.001). Resident clinic patients were more likely to carry a diagnosis of heart failure (*p* = 0.045). Further, only 15.7% of resident clinic patients were seen by any pulmonologist in the year prior to their index resident clinic visit. 

Patients in the post-intervention resident clinic cohort were more likely to have a lower FEV1/FVC (*p* = 0.04), lower FEV1 (*p* = 0.005), higher total lung capacity (*p* = 0.001), and more likely to be prescribed a long acting muscarinic agent (*p* = 0.004) when compared to the pre-intervention residents’ clinic cohort. The baseline characteristics of these cohorts are shown in Table 1. 

Prior to intervention, patients with a clinical diagnosis of COPD in the residents’ clinic had office spirometry or PFTs in 66.5% of cases, with 42.6% of these tests showing no airway obstruction as defined by Global Initiative for Chronic Obstructive Lung Disease (GOLD) guidelines [2]. This performance was significantly inferior to the patients seen in the fellows’ clinic where 83.6% had spirometry or PFTs (*p* < 0.001) with 23.8% (*p* < 0.001) of these having no obstructive airway disease. Residents provided smoking cessation counseling to 81.8% of active smokers while fellows counseled 91.6% of active smokers (*p* = 0.024). There was no significant difference in the rate of pneumococcal vaccination between the two clinics. These results are illustrated in Table 2.

After QI intervention, 119 subsequent visits to the residents’ clinic in a single academic year were analyzed. Active smokers received cessation counseling in 86.8% of the visits compared to 81.8% prior to QI intervention (*p* = 0.360). After the QI intervention, 74.8% of patients who carried a diagnosis of COPD underwent spirometry or PFTs, improved from 66.5% in the pre-intervention cohort (*p* = 0.085). Pneumococcal vaccination rates significantly improved to 72.3% from 61% pre-intervention (*p* = 0.024). These results are seen in Table 3.

## 4. Discussion

This study shows a significant improvement in the rates of pneumococcal vaccination after the implementation of a QI regimen directed at internal medicine residents by their peers. Improvements in COPD patients who had spirometry and COPD patients who received smoking cessation counseling were seen but were statistically non-significant. The initial retrospective phase suggests internal medicine residents did not perform as well as pulmonary fellows in adherence to guideline-based COPD care measures. While this finding may not be true across all United States-based training programs, it is consistent with studies showing more inconsistent COPD guideline adherence among primary care providers when compared to pulmonary specialists [18,19,20,21]. Rates of smoking cessation counseling and the utilization of spirometry in diagnosis of COPD improved in a non-significant fashion, as well. 

Successfully using residents as vehicles for QI has been reported elsewhere [22]. Our three specific quality improvement measures, namely smoking cessation in active tobacco users, pneumococcal vaccination, and diagnosis by spirometry, were chosen based on clinical importance, poor adherence noted in our residents’ clinic compared to the pulmonology fellows’ clinic, and the feasibility of improvement in a single QI cycle. Our resident-led strategy involved a multifaceted approach of a short presentation during a weekly resident didactic session, ongoing dissemination of study results to keep residents aware of the study and informed on deficiencies, posting index cards noting pneumococcal vaccination guidelines at each workstation in the clinic, and implementing a standing order for pneumococcal vaccination for patients with a diagnosis of COPD. This strategy was chosen because they were seen as the most efficient means by which the residents performing the QI study could reach their peers with the limited resources available. 

Several QI strategies in the literature have been described to improve the rates of pneumococcal vaccinations [23,24,25]. One study described a simple physician reminder document as sufficient to produce a significant improvement in pneumococcal vaccination in ambulatory rheumatology clinics. This particular trial quoted an improvement from a 67.6% to 80% vaccination rate, which was similar to our improvement from 61% to 72.3% post-intervention [23]. Our use of reminder cards mirrored the intervention in this study [23], and demonstrates simplistic interventions are useful at creating meaningful, statistically significant change. Further, involving clinic staff by means of a standing order decreased the chance of pneumococcal vaccination being forgotten during a clinic encounter. 

Our intervention focused primarily on improving resident knowledge of guidelines as they pertain to spirometry being used in the diagnosis of COPD [2]. It has been suggested that a “chronic care model” that involves both the patient and non-physician staff members actively seeking to improve access to spirometry may be the most effective approach to improve rates of spirometry in patients with COPD symptoms [12]. Educational endeavors directed at providers (similar to our didactic talk and e-mail correspondence with residents) have been shown to improve appropriate application of spirometry in clinical practice [26]. While we do not demonstrate a significant improvement, the trend towards improvement in this facet of care may suggest a promising switch in clinic culture toward a guideline-based, objective assessment of pulmonary symptoms. To improve further, we would involve non-physician staff (e.g., nursing to identify who would benefit from bedside spirometry) as targets of QI intervention similar to the model purported by Joo et al. [12]. Anecdotally, the increased use of spirometry in patients with a diagnosis of COPD or pulmonary symptoms was not felt to improve the accuracy of COPD diagnosis in the residents’ clinic. An interesting follow-up PDSA cycle could focus on improving the accuracy of diagnosis of COPD in our clinic and finding root cause of failure to consider alternative diagnoses.

Residents have been shown to improve their approach to smoking cessation counseling after receiving formal training on smoking cessation techniques [27,28]. Our intervention involved no formal training and was tailored to data showing that brief physician advice alone was adequate to improve the 1-year smoking quit rate [29]. Residents improved, albeit non-significantly, in rates of smoking cessation counseling after quality improvement intervention (81.8% to 86%). Larger, significant improvements have been shown in formally-trained residents taking care of smokers with and without chronic lung disease (10% to 21%) [28]. Although our demonstrated improvement is not statistically significant, we found the numerical improvement to be potentially promising for the brevity and simplicity of our intervention. Endeavors to incorporate formal training into residency didactics concerning smoking cessation counseling and therapeutic approaches is a potential future direction.

Among the limitations of our approach, we do not know the number of patients who were referred to the clinics with an incorrect diagnosis of “COPD” from a hospital admission and treated as such. Although this study was single center, internal medicine training programs in the United States may be able to implement these changes and replicate the results, due to their simplicity. However, every aspect of residency training is not uniform, which makes complete generalizability of this single-center study unlikely. Because a major goal of this QI project was to educate residents on guideline-based COPD care and improve clinician-in-training approach to patients with COPD, we elected not to have a control group who did not undergo QI intervention. Further, durability over time with this intervention is not presented, nor does the data extend beyond a single PDSA cycle. As our initial intervention only focused on spirometry being performed and not the result, a subsequent PDSA cycle focused on spirometry analysis to improve the accuracy of COPD diagnosis and adherence to treatment guidelines would be the natural next step. Calling attention to the use of spirometry could potentially lead to over-prescription of this diagnostic tool. Data is not available on the change in the clinic-wide use of spirometry based on this QI intervention. Also, prospective data in the fellows’ clinic was not collected, which may introduce bias to the study. As this study was performed as an uncontrolled before-after design, a longer trial would have granted more statistical power to assess smoking quit rate, and provide more insight into COPD exacerbation rate and all-cause hospitalization, allowing for a more robust analysis of patient outcomes.

## 5. Conclusions

In conclusion, this study demonstrated a resident-led QI intervention directed towards peers. It illustrated a succinct and low-cost method of education that was successful at eliciting a change in approach to guideline-based care of COPD patients. Statistical improvement in the number of patients who received pneumococcal vaccination and non-significant improvement in patients diagnosed with COPD who underwent spirometry and smoking cessation was noted. Further, it demonstrated the continued educational strides needed to improve care of chronic lung disease in general internal medicine training.

## Figures and Tables

**Table 1:** Baseline clinical characteristics of cohorts.

	Fellows’ Clinic, (*n* = 177)	Pre-Intervention Residents’ Clinic, (*n* = 451)	Post-Intervention Residents’ Clinic, (*n* = 119)
Age, mean (SD)	57.07 (8.23)	58.9 (9.1)	58.7 (8.6)
Female, *n* (%)	101 (57.06) *	216 (47.9)	55 (46.2)
Physician-documented history of asthma, *n* (%)	21 (11.86)	63 (14.1)	21 (17.7)
Physician-documented history of congestive heart failure, *n* (%)	28 (15.82) *	104 (23.2)	23 (19.3)
FEV1/FVC, mean (SD)	56.5 (16.2) *	63.4 (15.6)	60.1 (15.4) *
FEV1, % predicted, mean (SD)	54.8 (22.8) *	64.2 (23.4)	57.4 (23.1) *
Total Lung Capacity, % predicted, mean (SD)	97 (22.7)	94 (21.7)	102.3 (24.6) *
Residual Volume, % predicted, mean (SD)	128.4 (51.5) *	116 (48.8)	123.3 (48.2)
Diffusion Capacity, % predicted, mean (SD)	58.8 (20) *	65.4 (21.3)	66.5 (23.7)
Use of short acting beta-agonist, *n* (%)	158 (89.27) *	396 (88)	106 (89.1)
Use of long acting beta-agonist, *n* (%)	134 (75.71) *	260 (57.8)	75 (63)
Use of inhaled corticosteroid, *n* (%)	141 (79.66)*	291 (64.7)	79 (66.4)
Use of long-acting muscarinic antagonist, *n* (%)	92 (51.98)*	162 (36)	60 (50.4) *
Active tobacco smoker, *n* (%)	95 (54.9)	252 (58.3)	67 (56.3)
Home oxygen, *n* (%)	47 (26.2) *	64 (14.2)	21 (17.7)
Patients seen by a pulmonologist in the year prior to index visit, *n* (%)	177 (100) *	70 (15.7)	25 (21)

* Indicates significant (*p* < 0.05) difference when compared to pre-intervention resident’s clinic.

**Table 2:** Comparison of quality measure adherence in the pre-QI resident clinic and the fellows’ clinic.

	Residents’ Clinic, (*n* = 451)	Fellows’ Clinic, (*n* = 177)	*p* Value
Smoking cessation counseling documented, *n* (%) *	206 (81.8)	87 (91.6)	0.024
Spirometry performed, *n* (%)	300 (66.5)	148 (83.6)	<0.001
Obstruction confirmed by spirometry, *n* (%)	166 (36.8)	109 (61.6)	<0.001
Pneumococcal vaccine, *n* (%)	271 (61)	110 (62.5)	0.74

* For those who are active smokers.

**Table 3:** Result of QI intervention of quality measure adherence in residents’ clinic.

	Pre-Intervention	Post-Intervention	*p* Value
Spirometry performed, *n* (%)	300 (66.5)	89 (74.8)	0.085
Smoking cessation counseling documented, *n* (%) *	207 (81.8)	58 (86.6)	0.360
Pneumococcal vaccination, *n* (%)	271 (61)	86 (72.3)	0.024

* For those who are active smokers.
